# Sanity and Insanity

**Published:** 1891-03

**Authors:** 


### Sanity and Insanity.

According to Dr. Geo. M. Beard the symptoms of sanity are as follows:

I. Activity of the instinct of self-preservation.

II. Adaption to environment.

III. Correspondence of character to age and station.

IV. Rememberable conscience.

The symptoms of insanity appear in the following order, the later acquisitions first disappearing and then the earlier:

I. There is a decline in manners, that is, minor morals; then more extensive moral decline.

II. Decline in the power of originating thought.

III. Decline in the power of acquiring thought.

IV. Decline in the memory of recent events.

V. Decline in the memory of old events.

Insanity has been defined as "a disorder in the power of adjustment of the organism to its environment."

